# Therapeutic Potential of Qilianxiaopi Formula: Targeting ADAM17-Mediated Chronic Inflammation in Atrophic Gastritis

**DOI:** 10.3390/ph18030435

**Published:** 2025-03-19

**Authors:** Sijing Du, Tianxiang Wang, Zhiqiang Li, Ting Li, Zelong Miao, Yuling Chen, Songbiao Zhu, Wei Wei, Haiteng Deng

**Affiliations:** 1 MOE Key Laboratory of Bioinformatics, Center for Synthetic and Systematic Biology, School of Life Sciences, Tsinghua University, Beijing 100084, China; ddusijing@163.com (S.D.);; 2 Wangjing Hospital, China Academy of Chinese Medical Sciences, Beijing 100102, China; 3 Chinese Institutes for Medical Research (CIMR), Beijing 100069, China

**Keywords:** chronic atrophic gastritis, Qilianxiaopi formula, network pharmacology, thermal proteome profiling, bio-layer interferometry, ADAM17

## Abstract

**Background**: Gastric cancer (GC) is a leading cause of mortality worldwide, particularly in China. Chronic atrophic gastritis (CAG) and intestinal metaplasia (IM) are recognized as precancerous conditions contributing to GC development. Qilianxiaopi formula (QLXP), a traditional Chinese medicine (TCM), has demonstrated significant therapeutic effects on CAG and IM; however, its underlying mechanisms remain poorly understood. **Methods**: This study utilized chromatography-mass spectrometry to identify the major compounds in QLXP. Network pharmacology was used to predict the associated targets of these components. Thermal proteome profiling (TPP) pinpointed the potential binding proteins of QLXP, which were validated by bioinformatic analyses. Bio-layer interferometry (BLI) was used to analyze the interactions between QLXP and its key target proteins, thereby determining their binding components. Molecular docking predicted the binding modes between the components and proteins. **Results**: ADAM17 was identified as a key binding protein for QLXP. Further investigation revealed that QLXP inhibits the enzymatic activity of ADAM17, thereby reducing the secretion of the pro-inflammatory cytokine TNF-α, contributing to the anti-inflammatory properties of QLXP. BLI confirmed direct and reversible binding interactions between QLXP and ADAM17. Narirutin, isolated from the ADAM17 binding fraction, displayed the highest affinity for QLXP. **Conclusions**: This study highlights ADAM17 as a key molecular target of QLXP and narirutin as its principal binding component. The integrated approach combining chromatography-mass spectrometry, network pharmacology, TPP, BLI, and molecular docking provides a robust framework for elucidating the mechanisms of action of TCM.

## 1. Introduction

Gastric cancer (GC) is one of the biggest contributors to the global cancer burden. Despite an overall drop in age-adjusted incidence, GC remains the fifth leading cause of cancer-related mortality worldwide [1]. High-income Asian Pacific and East Asian areas had the highest age-standardized incidence rates in 2017, with almost half of the worldwide cases appearing in China [2]. Chronic atrophic gastritis (CAG) and intestinal metaplasia (IM) are precancerous conditions that independently increase the risk of GC [3]. Studies have reported cumulative GC incidences of 2.4% at 10 years and 2% at 20 years in patients with IM [4] and higher incidences in those with extensive endoscopic atrophy or IM in Japan (5.3–9.8% at 5 years) [5]. Even after endoscopic resection, IM in the corpus can significantly increase the risk of metachronous gastric lesions [6]. 

*Helicobacter pylori* (*H. pylori*) is the most common risk factor for developing GC, due to inducing a chronic inflammatory environment. ADAM17 acts as a molecular switch, regulating inflammation and tissue regeneration, thereby participating in the inflammation–cancer transition [7]. In addition to the eradication of *H. pylori*, endoscopic surveillance is considered the primary approach for patients with CAG or IM, especially in the advanced stages of atrophic gastritis [3]. Traditional Chinese medicine (TCM) has also shown clinical advantages in treating CAG and IM, it alleviates clinical symptoms as well as gastric mucosal lesions [8,9,10]. Banxia Xiexin Decoction is a traditional prescription used to treat epigastric distension by employing the “Xinkai Kujiang” approach, which involves the regulation of gastrointestinal functions with pungent and bitter medicinal herbs [10]. Given that chronic atrophic gastritis (CAG) is characterized by glandular atrophy and microvascular circulation impairment [11], we have modified the original formula by adding herbs that nourish qi and promote blood circulation to develop the Qilianxiaopi (QLXP) formula, thereby aligning more closely with the pathological changes of CAG. In clinical terms, our randomized controlled trial (RCT) showed that QLXP mitigates gastric mucosal atrophy and IM. Mechanistically, preliminary in vitro studies revealed that QLXP effectively inhibits the epithelial–mesenchymal transition (EMT) process.

Traditional Chinese herbal formulae often contain complex mixtures of bioactive compounds that simultaneously target multiple cellular pathways, contributing to holistic therapeutic effects [12]. However, the complexity of herbal formulae makes it challenging to identify their specific targets and mechanisms of action. This study therefore aimed to identify target proteins and interactive components of QLXP, aligning with the characteristics of TCM. 

Network pharmacology is an emerging field that applies the principles of complex system biology to investigate the therapeutic mechanisms underlying TCM. It focuses on the intricate interactions between drugs and biological systems, especially at the molecular level, to predict potential targets and identify key nodes in drug-target-pathway networks [13,14]. To experimentally verify these predictions, thermal proteome profiling (TPP) offers a proteome-wide screening strategy. Based on the principle that ligand-bound proteins exhibit enhanced thermal stability, TPP subjects cell lysates to a temperature gradient and quantifies protein denaturation via mass spectrometry [15]. This technique has proven effective in identifying targets for diverse molecules, from cofactors to clinical drug candidates [16]. The integration of TPP with the cellular thermal shift assay (CETSA), which evaluates protein solubility in response to temperature changes, allows for a more rigorous validation of the temperature-dependent stabilization of target proteins [17,18]. Bio-layer interferometry (BLI) offers a sensitive and label-free approach for real-time biomolecular interaction analysis. By immobilizing target proteins, BLI enables the identification of binding partners through a process known as “affinity fishing” [19]. Molecular docking, a computational technique, simulates the binding modes and affinities of small molecules (ligands) with biological macromolecules (targets), facilitating the identification of the most promising drug candidates based on their predicted interactions [20]. The synergistic application of network pharmacology, TPP, BLI, and molecular docking provides a comprehensive framework for investigating the target proteins of QLXP. This integrated approach promises to yield insights into the molecular underpinnings of QLXP’s therapeutic efficacy, thereby advancing our understanding of its mechanisms of action. 

## 2. Results

### 2.1. Prediction of the QLXP Potential Targets Using Network Pharmacological Analysis

Utilizing UPLC-Orbitrap MS, we characterized the phytochemical composition of QLXP’s aqueous extract. To pinpoint the primary constituents driving its therapeutic benefits, we applied two selection criteria: (1) We employed the “mzVault Best Match” over 80 to ensure reliable identification and reduce false positives. (2) We calculated the “Sample/Control Ratio” to assess the differential abundance in QLXP relative to placebo, focusing on compounds with a ratio exceeding 20. After data filtering, we identified 32 components in negative ion mode (Table 1) and 30 in positive ion mode (Table 2.), resulting in a consolidated list of 54 unique compounds after deduplication.

**Figure 1:**
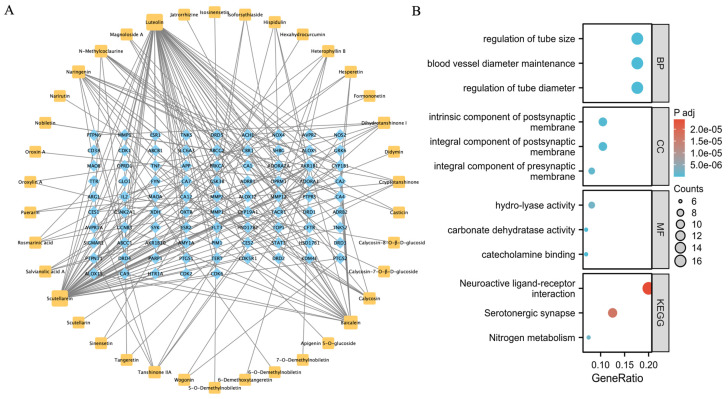
Network pharmacology analysis of QLXP. (**A**) The network of main components and predicted targets. Orange round rectangles represent components and blue diamond nodes represent relevant targets. (**B**) GO enrichment analysis and KEGG pathway enrichment analysis of predicted targets.

In the Swiss Target Prediction database (http://swisstargetprediction.ch/, accessed on 12 May 2023), the targets for the 54 unique compounds were predicted and filtered based on a probability value threshold of ≥0.3, resulting in 86 targets corresponding to 38 components. These association data were then imported into Cytoscape 3.8.0 (https://cytoscape.org/, accessed on 15 May 2023) to generate a component-target network comprising 124 nodes and 225 edges (Figure 1A). The results of the GO enrichment analysis showed that the mechanisms of action of QLXP were mainly related to the regulation of the tube, blood vessel diameter maintenance, integral components of the postsynaptic membrane, and hydrolyase activity, whereas the results of the KEGG pathway analysis indicated that QLXP is involved in neuroactive ligand–receptor interactions, the serotonergic synapse, and nitrogen metabolism pathways (Figure 1B).

### 2.2. Identification of QLXP Targets Using Thermal Proteome Profiling

**Figure 2:**
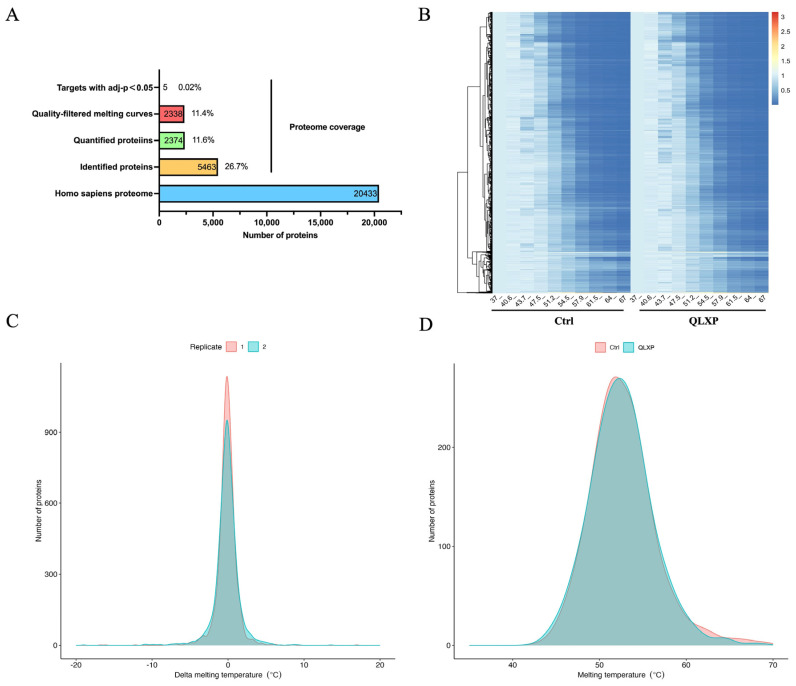
Comprehensive analysis of the thermal proteome profiling experiment for QLXP in GES-1 cell lysates. (**A**) Statistical numbers of TPP data compared to the proteome of *Homo sapiens*. (**B**) The representative heatmap of the non-denatured fraction under different temperatures of all proteins, which reflected the global precipitation behavior of proteins after heat treatment in control or QLXP-treated lysates. The colors suggest protein abundance levels of the non-denatured protein fractions after heat treatment at one of ten temperatures. Relative abundances were normalized to the abundance at 37 °C. (**C**) Tm differences (ΔTm) of proteins in two biological replicates are reproducible. (**D**) TPP experiments captured a large dynamic range of proteins and depicted the distribution of melting temperatures (Tm) in GES-1 cells.

**Figure 3:**
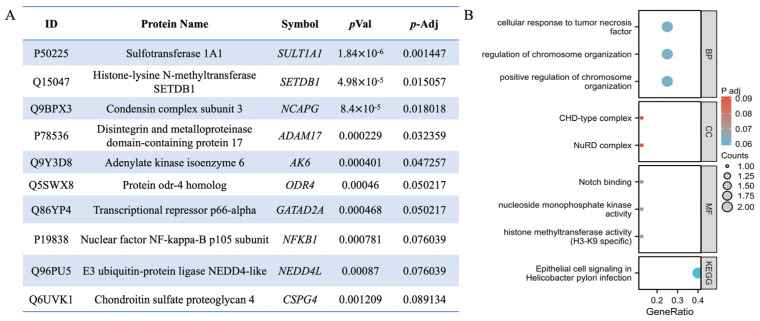
(**A**) The list of 10 prime targets with adjusted *p*-value < 0.1. Fstat computed with NPARC method quantifies the relative reduction in residuals from null to alternative model. *p* values were computed from the values of the Fstat, and the Benjamini–Hochberg procedure was used to adjust the *p* values for multiple hypothesis test. (**B**) GSEA of these 10 proteins.

**Figure 4:**
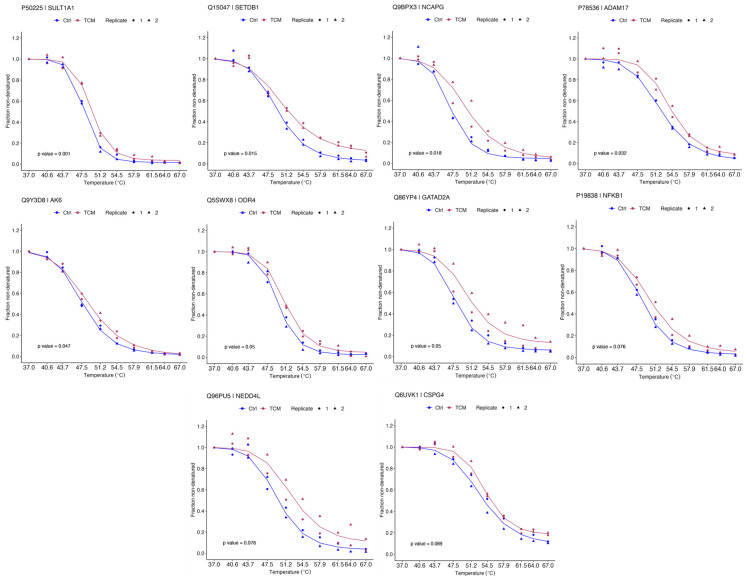
The melting curves of 10 prime targets with adjusted *p* values < 0.1. TCM stands for Traditional Chinese Medicine, specifically referring to the QLXP.

The TPP analysis revealed 5463 proteins across biological duplicates, of which 2374 were successfully quantified. After using a filtering criterion, 2338 proteins produced valid sigmoidal melting curves (Figure 2A), permitting the determination of melting temperature (Tm), which is the point at which half of the protein is denatured. After evaluating the reproducibility of our TPP-based QLXP assessment, we observed a robust Pearson’s correlation coefficient between 0.83 and 0.93 for Tm values across biological replicates, indicating high consistency. Overall, the protein abundance decreased as the temperature increased (Figure 2B). The distribution of the Tm in the human proteome ranged from 45–70 °C (Figure 2C). To investigate the impact of QLXP on protein stability, we calculated the ΔTm (TmQLXP-TmCtrl) for each protein (Figure 2D). Using NPARC analysis to minimize false negatives, we pinpointed 263 potential interactors with statistical significance (*p* < 0.05), including 115 proteins that exhibited a marked QLXP-mediated shift (*p* < 0.01) in thermal stability. After applying a more rigorous threshold of adjusted *p* < 0.1, we identified 10 proteins as prime QLXP targets (Figure 3A). The gene set enrichment analysis (GSEA) revealed that ADAM17 and NF-κB1 are closely associated with cellular responses to tumor necrosis factor and *H. pylori* infection (Figure 3B). The melting curves of 10 prime targets with adjusted *p* values < 0.1 are shown in Figure 4.

### 2.3. ADAM17 Is a Key Candidate Target Associated with CAG and GC

**Figure 5:**
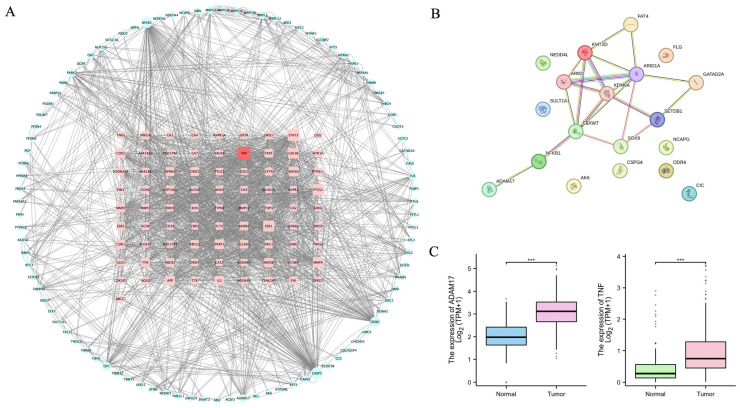
Bioinformatic analysis of predicted targets, binding targets, and public data. (**A**) Protein–protein interaction network of predicted targets and binding targets with QLXP. Blue circles represent binding targets with QLXP, pink round rectangles represent predicted targets. Node size indicates the degree of connectivity, with larger nodes representing higher connectivity. TNF is the top node. (**B**) Protein–protein interaction analysis between the proteins of nine IM driver genes and QLXP targets with adjust-*p* < 0.1. (**C**) The expression of ADAM17 and TNFα in GC and normal tissue of TPM-formatted RNA-seq data from TCGA and GTEx datasets, Statistical significance is indicated as follows: *** *p* < 0.001.

We conducted a joint analysis of the above network pharmacology and TPP results and integrated relevant literature reports to identify key targets for subsequent research. We combined the 86 targets derived from network pharmacology with the 115 significant QLXP-modulated proteins from TPP and imported them into STRING for Protein–Protein Interaction (PPI) analysis, specifying the *Homo* species. Following the elimination of proteins without connections, we rendered the network in Cytoscape 3.8.0, consisting of 177 nodes and 1702 edges (Figure 5A). By calculating the degree parameter, TNF was identified as the top target. Furthermore, we evaluated a prospective multicenter longitudinal cohort study [21], in which researchers analyzed 1256 gastric samples and identified 26 IM driver genes across different pathways, including 9 significant driver genes. We also performed a PPI analysis between the proteins of these nine significant IM driver genes and QLXP targets with adjusted *p* < 0.1 from TPP, revealing a notable interaction between ADAM17 and the driver genes, mediated by NF-κB1 (Figure 5B). 

Chronic inflammation is closely related to CAG. Through the aforementioned combined analysis, it so happens that TNF, ADAM17, and NF-κB1 are all important molecules associated with chronic inflammation. ADAM17, also known as TNF-α-converting enzyme (TACE), is crucial for the cleavage and release of TNF-α from its membrane-bound precursor. This process allows TNF-α to bind to its receptors and initiate the classical NF-κB inflammatory pathway, leading to pro-inflammatory effects [22]. NF-κB transcription factors play a role in the regulation of ADAM17 expression [23]. Given this interplay between ADAM17 and the inflammatory cascade, selecting ADAM17 as a pivotal research target protein is strongly justified. Moreover, since NF-κB1 has been extensively studied in CAG, we intend to focus our research on ADAM17. To delve into the relationship between ADAM17 and gastric mucosal inflammation-related cancer, we analyzed public databases, including TPM-formatted RNA-seq data from the TCGA and GTEx datasets, to investigate the transcriptional variations between GC and normal tissues. These datasets were uniformly processed using the Toil pipeline (https://toil.ucsc-cgl.org/, accessed on 20 July 2023) [24] and obtained from the UCSC XENA database (https://xenabrowser.net/datapages/, accessed on 20 July 2023). Our analysis included 414 GC and 210 normal tissue samples. Upon comparing the transcriptional profiles, we found that ADAM17 and TNF-α mRNA levels were significantly increased in GC tissues compared to those in normal tissue (Figure 5C). We therefore considered ADAM17 to be a key candidate target associated with CAG and GC.

### 2.4. Validation of ADAM17 as a Target of QLXP

**Figure 6:**
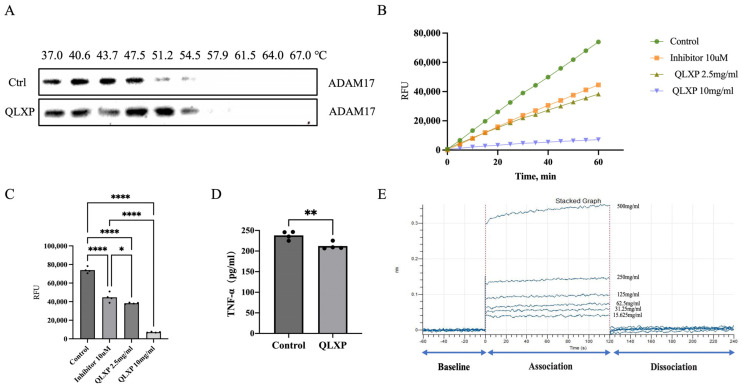
(**A**) CETSA of ADAM17 with escalating temperature by Western blotting. (**B**) Kinetics analysis of the inhibitory effect of QLXP on the enzymatic activity of ADAM17. (**C**) Endpoint analysis of the inhibitory effect of QLXP on the enzymatic activity of ADAM17. Statistical significance is indicated as follows: * *p* < 0.05, **** *p* < 0.0001. (**D**) Inhibitory effect of QLXP on the expression of TNF-α. ** *p* < 0.01. (**E**) Real-time kinetic binding sensorgrams of different concentrations of QLXP increasing from 15.625 to 500 mg/mL are shown. Response (nm) indicates the optical thickness on the SAA biosensor layer.

To confirm the interaction between QLXP and ADAM17, we conducted CETSA to assess the temperature-dependent alterations in ADAM17 stability. Under experimental conditions analogous to TPP, ADAM17 exhibited increased stability in the presence of QLXP, confirming its role as an QLXP-binding partner (Figure 6A).

To investigate whether QLXP attenuates the enzymatic activity of ADAM17, we incubated GES-1 cell lysates with H_2_O as negative control and ADAM17‘s inhibitor as positive control, subsequently assessing changes in ADAM17 activity using an ADAM17 activity assay kit (AnaSpec, Fremont, CA, USA; cat. #AS-72085). Following dual analysis of kinetic and endpoint data, we found that QLXP significantly inhibits the enzymatic activity of ADAM17 (Figure 6B,C). 

As ADAM17 functions as TACE and AGS cells express abundant TNF-α, to assess whether QLXP inhibits the expression of TNF-α, we treated AGS cells with QLXP for 12 h, using H_2_O as a negative control. We found that QLXP exerted an inhibitory effect on the release of TNF-α (Figure 6D). In summary, the integrated analysis of the interactions between QLXP and ADAM17, along with the assessment of its influence on TNF-α, elucidated the molecular basis of the therapeutic efficacy of QLXP.

### 2.5. BLI Analysis Identifies Components Binding to ADAM17 in QLXP

BLI provides a sensitive and label-free method for real-time analysis of biomolecular interactions. We initially employed BLI to evaluate the binding of QLXP to ADAM17, monitoring the real-time association and dissociation kinetics between QLXP (at concentrations from 15.625 to 500 μg/mL) and biotinylated ADAM17. The findings revealed a clear, concentration-responsive, and reversible interaction between the two molecules, as evidenced by the proportional increase in the optical thickness (nm) measured on the sensor surface (Figure 6E). In summary, using TPP, CETSA, and BLI analyses, we confirmed that ADAM17 is a binding partner of QLXP.

To identify the components in QLXP that interact with ADAM17, we collected the dissociation solutions from both the QLXP group and the blank control group for analysis using UPLC-Orbitrap MS. For the identification of ADAM17-binding components, we applied two specific criteria: (1) The “mzVault Best Match” score must have exceeded 70. (2) Not present in the dissociation solution of the blank control group. After the rigorous quality control of signal stability, we identified the components that bind to ADAM17 (Table 3). Among the screened components, alkaloids and flavonoids were most abundant, followed by isoflavones and organic acids. Alkaloids and flavonoids, two types of plant secondary metabolites, have various biological properties, including antioxidant, anti-inflammatory, antiviral, anticancer, antiplatelet, and antibacterial properties.

### 2.6. Molecular Docking Analysis of ADAM17 and Its Binding Components

**Figure 7:**
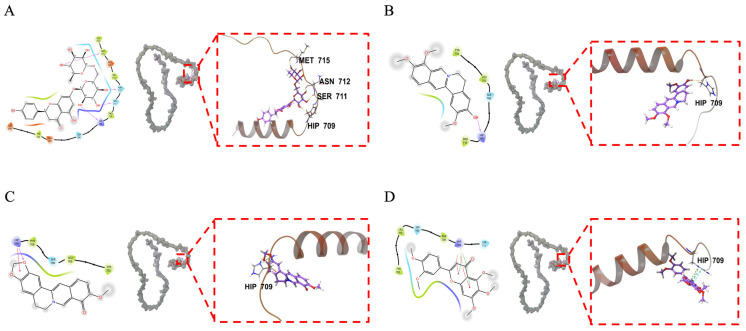
Molecular docking result of narirutin, jatrorrhizine, berberrubine, and sinensetin with ADAM17. (**A**) Docking diagrams of narirutin and ADAM17 protein. Narirutin binds to the surface of the active pocket in the intracellular domain of ADAM17 protein, forming a hydrogen bond with each of the residues HIP709, SER711, ASN712, and MET715 in the ADAM17 protein. Yellow represents hydrogen bonding. (**B**) Docking diagrams of jatrorrhizine and ADAM17 protein. Jatrorrhizine binds to the surface of the active pocket in the intracellular domain of the ADAM17 protein, forming a hydrogen bond with the residue HIP709 in the ADAM17 protein. Yellow represents hydrogen bonding. (**C**) Docking diagrams of berberrubine and ADAM17 protein. Berberrubine binds to the surface of the active pocket in the intracellular domain of the ADAM17 protein, forming a hydrogen bond and a π–cation bond with the residue HIP709 in the ADAM17 protein. Yellow represents hydrogen bonding, while green represents π–cation bonding. (**D**) Docking diagrams of sinensetin and ADAM17 protein. Sinensetin binds to the surface of the active pocket in the intracellular domain of the ADAM17 protein, forming two π–π bonds and two π–cation (π-positive ion) bonds with the residue HIP709 in the ADAM17 protein. Blue represents π –π bonds, while green represents π–cation (π-positive ion) bonds.

Based on the binding component classification, we selected two alkaloids (jatrorrhizine and berberrubine) and two flavonoids (narirutin and sinensetin) for molecular docking analysis. The XP Gscore is commonly used as a reference metric. A binding free energy is considered low when the XP Gscore is below −6 or the MM-GBSA dG Bind value is less than −30 kcal/mol, signifying a stable interaction between the ligand and the protein [25]. Our analyses revealed that all four components exhibited significant binding affinity towards ADAM17. Notably, narirutin and berberrubine showed promising interactions with ADAM17, which exhibited the best docking performance, indicating particularly stable binding with ADAM17 (Table 4). In detail, narirutin formed hydrogen bonds with HIP709, SER711, ASN712, and MET715 (Figure 7A). Similarly, jatrorrhizine also bound to HIP709 via a hydrogen bond (Figure 7B). Berberrubine interacted with HIP709 via both a hydrogen bond and a π–cation bond (Figure 7C). Sinensetin formed two π–π bonds and two π–cation bonds with the same residue (Figure 7D). These findings offer critical insights into the molecular interactions, elucidating the specific binding modes and the potential for these components to modulate ADAM17 activity. The predominance of HIP709 in the binding events suggests that this histidine residue acts as a pivotal anchor point within the ADAM17 catalytic domain. Interactions between ligands and HIP709, such as hydrogen bonds and π–cation interactions, may inhibit enzyme activity. Adjacent residues, such as SER711, ASN712, and MET715, are likely involved in the stability of the binding pocket. 

## 3. Discussion

CAG, a chronic inflammatory condition characterized by the progressive loss of gastric glandular cells, is considered a precancerous lesion and is strongly correlated with an increased risk of GC. Our prior RCT revealed that QLXP can alleviate gastric mucosal atrophy and IM, and it effectively inhibits the EMT process. However, the molecular targets of QLXP remained undefined. By integrating network pharmacology, TPP, BLI, and molecular docking, the target proteins and interacting compounds of QLXP were identified in the present study. 

We utilized UPLC-Orbitrap MS to analyze the aqueous extract of QLXP, identifying 54 unique compounds after applying strict criteria. Targets for these compounds were predicted using the Swiss Target Prediction database, resulting in 86 targets linked to 38 components. Subsequently, we used TPP to identify 115 potential targets of QLXP. A PPI analysis of the 86 predicted targets and 115 significant QLXP-shifted proteins identified TNF as the top target among the predicted targets. Through the implementation of a rigorous data filter in TPP, 10 proteins, including ADAM17, were found to bind to QLXP with high confidence. After considering the relationship between ADAM17 and TNF, the association with IM driver genes, and differential expression in GC tissues, ADAM17 was determined to be a key candidate target of QLXP associated with CAG and GC. Therefore, we verified the binding of QLXP to ADAM17 using CETSA and BLI techniques.

ADAM17 belongs to the “A Disintegrin And Metalloproteinase” (ADAM) family of type I transmembrane proteins [26,27]. ADAM17 is primarily involved in the ectodomain shedding of membrane proteins, including cytokines, growth factors, receptors, and adhesion molecules, and plays a role in cell signaling, adhesion, migration, and other biological processes [7]. ADAM17 further plays a pivotal role in inflammation and cancer, as ADAM17-mediated shedding events have been implicated in regulating inflammatory responses and promoting tumor growth, invasion, and metastasis [27,28,29,30]. ADAM17 mediates the proteolytic cleavage and release of inflammatory mediators, such as TNF-α and IL-6R, in gastric mucosal inflammation. During *H. pylori* infection, CagL dissociates ADAM17 from integrin α5β1, activating the ADAM17-dependent and NF-κB-mediated repression of HKα, intensifying the inflammatory process [31,32]. Furthermore, ADAM17 facilitates EMT in gastric cancer cells by activating the TGF-β/Smad signaling pathway, thereby contributing to tumor progression and metastasis [33]. Beyond inflammation and cancer, ADAM17 has been implicated in the pathogenesis of other diseases. In cardiovascular diseases, it may contribute to atherosclerosis and heart failure through the shedding of cell adhesion molecules [34]. In neurological disorders, ADAM17 could be involved in the cleavage of neuregulin, affecting synaptic function and contributing to diseases like Alzheimer’s and Parkinson’s [35]. In autoimmune diseases, the dysregulation of ADAM17 activity may lead to an imbalance in immune responses, exacerbating conditions such as rheumatoid arthritis and multiple sclerosis [36]. ADAM17 is a pivotal enzyme in the elucidation of disease pathogenesis and serves as a promiscuous therapeutic target in pharmacological research. Our findings reveal that QLXP effectively inhibits the enzymatic activity of ADAM17, thereby decreasing the secretion of TNF-α. This mechanism of action clarifies QLXP’s inhibitory effect on gastric mucosal inflammation. These results provide preliminary evidence supporting the potential development of QLXP as an ADAM17 inhibitor. However, the development of ADAM17 inhibitors requires addressing challenges such as precise delivery, dynamic monitoring, and combination therapy to overcome issues of toxicity and resistance, thereby facilitating their transition to clinical application [37].

Other potential targets of QLXP include enzymes (SULT1A1 and AK6), transcription factors (SETDB1 and GATAD2A), signal transduction proteins (ODR4), cell surface proteoglycans (CSPG4), chromatin remodeling complex components (NCAPG), and ubiquitin ligases (NEDD4L). While these targets were identified, the current study did not focus on their specific interactions with QLXP. Nonetheless, the potential involvement of these molecules warrants further discussion. SETDB1’s role in epigenetic regulation and its association with GC suggest that it could be a significant player in the therapeutic response to QLXP. Future work could explore the extent to which QLXP modulates SETDB1 activity and its downstream effects on gene expression in the context of gastric mucosal inflammation and cancer.

BLI has played a crucial role in screening and validating the binding affinities of small molecules from TCM to Aβ peptides, which are key pathological features of Alzheimer’s disease [38,39]. Through BLI technology and affinity-fish compounds binding ADAM17 in QLXP, we identified a range of bioactive compounds, including alkaloids, flavonoids, isoflavones (formononetin and dehydroglaucine), and organic acids. After a molecular docking analysis, we found that narirutin exhibited the best docking performance with ADAM17, indicating stable binding between this compound and the intracellular domain of ADAM17. Narirutin is a flavonoid compound commonly found in citrus peels, such as the *Citrus × aurantium* L. in QLXP. Studies have demonstrated that narirutin has diverse pharmacological properties, such as antioxidant, anti-inflammatory, anticancer, and neuroprotective properties [40]. Further studies should be conducted to investigate the anti-inflammatory and anticancer effects of narirutin. In present study, narirutin may directly affect enzyme activity through HIP709. Adjacent residues, such as SER711, ASN712, and MET715, are likely involved in substrate recognition and the stability of the binding pocket. In future studies, we intend to utilize site-directed mutagenesis and other techniques to further validate these interactions and their impact on ADAM17 function. Additionally, berberrubine and sinensetin exhibited promising binding affinities. Berberrubine, the major metabolite of berberine, originates from *Coptis chinensis Franch* in QLXP. Previous studies have shown that berberrubine has pharmacological properties, including anti-tumor, anti-inflammatory, and lipid-lowering functions [41]. Sinensetin is a plant-derived polymethoxylated flavonoid found in citrus fruits, such as the *Citrus × aurantium* L. In vitro and in vivo studies have confirmed the potent anticancer effects of sinensetin, along with its broad pharmacological profile, which includes anti-inflammatory, antioxidant, anti-obesity, anti-dementia, and vasorelaxant activities [42].

In summary, the synergistic application of network pharmacology, TPP, BLI, and molecular docking provides a comprehensive framework for investigating the target proteins of QLXP. To date, the application of such a systematic methodological combination in the research of TCM is relatively rare. We are hopeful that our research methodology will gain recognition and be broadly applied in the field. However, it is crucial to acknowledge the limitations inherent in the current research design. The primary limitation of this study is its restriction to in vitro cellular experiments, which may not entirely reflect the intricate physiological and pathological dynamics within a living organism. To address this, we intend to confirm the anti-inflammatory properties of QLXP in a murine model of CAG, with a particular emphasis on the suppression of TNF-α and the modulation of ADAM17 activity in a systemic inflammatory context. Our investigation has been focused on the interaction between ADAM17 and narirutin, a single target and component of QLXP. However, QLXP’s therapeutic effects are likely multifaceted, involving multiple targets and constituents. The impact of narirutin on proteins other than ADAM17 remains to be explored. Consequently, further in vivo studies are necessary to corroborate our in vitro findings and to identify additional potential targets and active components of QLXP. Such research will deepen our understanding of QLXP’s therapeutic promise and its utility in the management of CAG and IM.Additionally, the study may be influenced by confounding factors, including the complexity of the herbal formulation, inter-individual variability, and dose-related effects, all of which can affect the medication’s efficacy. To overcome these challenges, a systems pharmacology approach is advocated, which incorporates AI for mapping component interactions, multi-omics for patient stratification, and physiologically-based pharmacokinetic modeling. This approach will not only enhance the robustness of our findings but also provide a more comprehensive understanding of QLXP’s mechanism of action and its potential clinical application. Ultimately, this method holds the potential to transform traditional herbal practices to a modern, mechanism-guided approach within the realm of Traditional Chinese Medicine.

## 4. Materials and Methods

### 4.1. Network Pharmacology

#### 4.1.1. Aqueous Extraction and Preparation of QLXP

QLXP granules were sourced from Guangdong Yifang Pharmaceutical Co., Ltd. (Foshan, China). The active ingredients in QLXP were extracted using the aqueous extraction method. One gram of QLXP granules was dissolved in 10 mL of distilled water, heated to 100 °C in a water bath for 1 h, and centrifuged to collect the supernatant. The supernatant was then filtered through a 0.22 µm filter in a sterile environment and stored at 4 °C for future use.

#### 4.1.2. LC-MS/MS Analysis

The aqueous extract of QLXP was analyzed using UPLC-Orbitrap MS. Separation was achieved on an ethylene bridged hybrid C18 column (Waters, Milford, MA, USA) with a binary solvent system (A: H_2_O + 0.1% formic acid; B: CAN + 0.1% formic acid) at 250 μL/min over 25 min. Data were acquired in both positive and negative ion modes with MS/MS and subsequently processed and identified using the Compound Discoverer 3.3 software. 

#### 4.1.3. Prediction of the Potential Targets of the Major Compounds in QLXP

The major components were converted into standard SMILES files using ChemSpider (https://www.chemspider.com/, accessed on 5 May 2023) [43]. The potential targets of the major compounds in QLXP were obtained from the Swiss Target Prediction database (http://swisstargetprediction.ch/, accessed on 12 May 2023) [44].

#### 4.1.4. Construction and Analysis of the “Component-Target” Network

The “component-target” network of QLXP was constructed using Cytoscape 3.8.0. The Network analyzer plugin in the Cytoscape software was used to calculate parameters, such as degree centrality.

### 4.2. Thermal Proteome Profiling

#### 4.2.1. Cell Culture

GES-1 and AGS cell lines, obtained from the cell bank of the Chinese Academy of Sciences, were cultured in DMEM and RPMI-1640 media (Wisent, Montreal, QC, Canada). The culture media were supplemented with 10% fetal bovine serum (PAN-Biotech, Aidenbach, Bavaria, Germany) and 1% penicillin/streptomycin (Wisent, Montreal, QC, Canada). The cells were maintained in an incubator set to 37 °C with 5% CO_2_.

#### 4.2.2. Sample Preparation for Thermal Proteome Profiling

The GES-1 cells were collected, washed thrice with phosphate-buffered saline (PBS, pH 7.4), and resuspended in lysis buffer (PBS containing a protease inhibitor cocktail and 1.5 mM MgCl_2_). The cell suspension was then mechanically disrupted using a glass grinding rod (50 strokes). The methodology employed for the TPP used an established protocol with multiplexed quantitative mass spectrometry-based proteomics to evaluate the thermal stability of proteins across the proteome [45]. In brief, two groups of crude cellular extracts were incubated with 5 mg/mL QLXP and water for 15 min, respectively, with water serving as the negative control. Following this, aliquots of the treated lysates were subjected to a temperature range of 37–67 °C for 3 min, followed by incubation at 25 °C for 3 min. Membrane- and DNA-bound proteins were extracted using 0.8% NP40 (Biodee, Beijing, China; cat. #74385) and 250 U/mL benzonase (Sigma, St. Louis, MO, USA; cat. #E1014) and protein aggregates were removed via ultracentrifugation. The protein pellet was air-dried after removing the supernatant and acetone, then redissolved in 200 μL of 8 M urea in PBS. Reduction/alkylation was conducted with 5 mM DTT at 55 °C for 45 min, followed by 12.5 mM IAM at room temperature in the dark for 30 min. Samples were diluted to 1.5 M urea in PBS, trypsin was added at a 1:40 ratio, and digestion was performed overnight at 37 °C. Digestion was terminated by adding 10% TFA to acidify to pH 2. Desalting was carried out using a Sep-Pak C18 cartridge. Peptides were dried with SpeedVac (Thermo Scientific, Waltham, MA, USA) and resuspended in 50 μL of 100 mM TEAB before TMT labeling for 1 h at room temperature. The labeling reaction was quenched with 5% hydroxylamine, and mixed peptide samples were diluted, acidified, and desalted. The samples were dried to less than 300 μL and fractionated by high-pH reverse-phase HPLC. Fractions were concatenated into 12 samples, dried, and resuspended in 40 μL of 0.1% formic acid for LC-MS/MS analysis.

#### 4.2.3. LC-MS/MS Analysis and Protein Identification and Quantification

The LC-MS/MS analysis was conducted using a previously described method [45]. The labeled peptides were subjected to LC-MS/MS on a UHPLC3000 system coupled to a Q Exactive HF-X MS (Thermo Scientific, Waltham, MA, USA) with a procolumn connected to a C18 column (Waters, Milford, MA, USA). Protein identification and quantification were performed using Proteome Discoverer 2.3 and the Homo sapiens database.

#### 4.2.4. Bioinformatic Analysis

Gene Ontology (GO) enrichment analysis was conducted using the R package clusterProfiler 4.1.1 to categorize and annotate the functional properties of the target genes, highlighting significant GO terms. Using the KEGG API, we retrieved the background gene set and performed enrichment analysis using clusterProfiler 4.1.1 to further uncover the enriched pathways in our target gene set, offering insights into their biological roles. Additionally, we constructed a protein–protein interaction (PPI) network using the STRING database (https://string-db.org/, accessed on 1 July 2023) and visualized it using Cytoscape 3.8.0, revealing key interaction hubs and pathways.

### 4.3. Western Blotting

The cells were lysed using RIPA lysis buffer (Beyotime, Shanghai, China, cat. #P0013K) supplemented with a protease inhibitor cocktail. Equal protein volumes were separated on a 12% sodium dodecyl sulfate-polyacrylamide gel and electroblotted onto a polyvinylidene difluoride membrane. Immunoblotting was performed using the primary antibody anti-ADAM17 (Abcam, Cambridge, UK; cat. #ab291073) and secondary antibody anti-rabbit horseradish peroxidase (HRP)-IgG (Cell Signaling Technology, Danvers, MA, USA;cat. #7074).

### 4.4. ADAM17 Activity Assay

The effects of QLXP on ADAM17 activity were determined using the ADAM17 activity assay kit (AnaSpec, Fremont, CA, USA; cat. #AS-72085) with H_2_O as negative control and ADAM17‘s inhibitor as positive control, according to the manufacturer’s protocol. In brief, GES-1 cells were washed with PBS, lysed using assay buffer containing Triton-X 100, and centrifuged to collect the cell lysate in the supernatant. The TACE substrate was diluted in assay buffer to create a working solution. The TACE-containing samples were then added to a microplate, followed by a TACE substrate solution, and the plate was gently shaken to mix the reagents. Fluorescence was measured at Ex/Em = 490 nm/520 nm, either continuously over time or as an endpoint reading after a specified incubation period. The experiments were repeated four times.

### 4.5. Enzyme-Linked Immunosorbent Assay of TNF-α

The effects of QLXP on the secretion of TNF-α were assayed using the Human TNF-α enzyme-linked immunosorbent assay (ELISA) Kit (Proteinch, Rosemont, IL, USA; cat. #KE00154), according to the manufacturer’s guidelines. In brief, the supernatant from AGS cell culture media, after a 12 h QLXP treatment, was harvested and centrifuged at 500× *g* for 5 min. Standards or samples were then gradient-diluted and sequentially incubated with detection antibody and HRP-conjugated secondary antibody. The optical density (OD) of each well was measured at 450 nm using an ELISA reader with a correction wavelength of 630 nm, if available, to ensure accurate readings.

### 4.6. BLI Analysis

The BLI analysis was performed using the ForteBio Octet^®^ RED96e system (Göttingen, Germany). The ADAM17 fusion protein (Proteintech, Rosemont, IL, USA; cat. #Ag32418) was prepared in PBS and at a concentration of 50 μg/mL and subjected to biotinylating with EZ-Link NHS-LC-LC-Biotin. Prior to immobilizing the biotinylated ADAM17 onto the SSA biosensor, the biosensor was pre-wetted with PBS to establish a baseline. The extracts of QLXP were meticulously diluted within a PBS solution that was fortified with 15% DMSO and 0.02% Tween 20, with a final volume of 200 μL per well. An equivalent volume of the same PBS solution was dispensed into separate wells to constitute the control group of without QLXP or without ADAM17 fusion protein. The experiment proceeded in a cyclic fashion with four basic steps: loading for 300 s, establishing a baseline for 60 s, association for 120 s, and dissociation for 120 s. Data acquisition and analysis were performed using the ForteBio Octet Data Acquisition and Analysis software, with background subtraction performed using a double-negative control for interaction signals. Using UPLC-Orbitrap MS, the dissociation and control buffers for the BLI analysis were analyzed as described in Section 4.1.2 to identify components that interact with ADAM17.

### 4.7. Molecular Docking Analysis

Given that our study focused on the intracellular region of ADAM17 involved in the BLI analysis, we utilized AlphaFold2 to predict the crystal structure of the intracellular domain of ADAM17 [46]. The predicted protein structures were processed using the Protein Preparation Wizard module in Schrödinger software 2024-2. The 2D SDF structure files of the compounds were processed using the LigPrep module in the Schrödinger software to generate all the possible 3D chiral conformations. In molecular docking, the XP mode incorporated flexible docking, enabling both the protein and ligand to adapt to their conformations, thereby offering the most sophisticated computational approach. A subsequent analysis using MM-GBSA served as a robust validation step, reinforcing the XP docking findings.

### 4.8. Statistical Analysis

R (version 4.3) and GraphPad Prism software (version 9.0) were used for statistical analysis and plotting. Specifically, TPP data were fitted with two competing models in the NPARC method: a null model which analyzed the relative protein abundances changes with temperature by using a single smooth function of temperature irrespective of the ligand treatment, and an alternative model that analyzed the relative protein abundances changes for the vehicle and treatment group, respectively. The sum of squared residuals (RSS), which was calculated as the deviations between the fitted model and observed data, were used to calculate the F-statistic to estimate the variability between groups and within groups. For each protein, a *p*-value was computed from the F-statistic based on the cumulative F-distribution. To account for multiple hypothesis testing, the Benjamini and Hochberg method was applied to adjust the *p*-values across all proteins. Differences were considered statistically significant if Student’s *t*-test yielded *p*-values less than 0.05. Other data are presented as mean ± standard error of the mean (SEM). Statistical comparisons across all groups were performed using either a one-way analysis of variance (ANOVA) test or an unpaired Student’s *t*-test.

## 5. Conclusions

Utilizing network pharmacology and TPP, we pinpointed ADAM17 as a target of QLXP, where QLXP inhibits the enzymatic activity of ADAM17, thereby suppressing TNF-α secretion and exerting anti-inflammatory effects. BLI confirmed the direct and reversible binding of QLXP to ADAM17, with narirutin exhibiting the highest binding affinity. Our findings demonstrate that the “network pharmacology—TPP—BLI—molecular docking” method is a powerful tool to reveal the mechanisms of action of traditional Chinese herbal formulae.

## Figures and Tables

**Table 1:** Main components of QLXP in negative ion mode.

No.	Name	Formula	*m*/*z*	RT [min]	mzVault Best Match	Sample/ Control Ratio
1	*N*-Acetylglutamic acid	C_7_H_11_NO_5_	188.05663	1.403	88.6	29.27
2	Forsythoside E	C_20_H_30_O_12_	461.16685	5.795	88.3	43.39
3	Hastatoside	C_17_H_24_O_11_	449.13039	5.972	84.3	40.19
4	Atractyloside A	C_21_H_36_O_10_	493.22952	6.266	90.5	40.27
5	Purpureaside C	C_35_H_46_O_20_	785.25233	7.471	90.2	50.63
6	Vicenin II	C_27_H_30_O_15_	593.15181	7.583	89.5	33.36
7	Dihydromorin	C_15_H_12_O_7_	303.05097	7.886	92	104.08
8	Isoforsythiaside	C_29_H_36_O_15_	623.19852	8.252	91.1	109.93
9	Magnoloside A	C_29_H_36_O_15_	623.19843	9.288	89.2	83.28
10	Apigenin 5-*O*-glucoside	C_21_H_20_O_10_	431.09853	9.289	89.2	29.35
11	Narirutin	C_27_H_32_O_14_	579.17223	9.721	90.9	43.45
12	Isoacteoside	C_29_H_36_O_15_	623.19834	9.724	88.6	109.99
13	Rosmarinic acid	C_18_H_16_O_8_	359.07738	10.035	82.8	97.41
14	Salvianolic acid A	C_26_H_22_O_10_	493.11453	10.062	89.3	31.69
15	Puerarin	C_21_H_20_O_9_	415.10377	10.097	84.2	85.09
16	Hesperetin	C_16_H_14_O_6_	301.07183	10.225	92.8	40.93
17	Scutellarin	C_21_H_18_O_12_	461.07282	10.605	84.9	32.96
18	Baicalein	C_15_H_10_O_5_	269.04536	10.82	91	20.02
19	Oroxin A	C_21_H_20_O_10_	431.09846	11.188	88.1	35.62
20	Dalbergioidin	C_15_H_12_O_6_	287.05634	11.239	84.8	150.86
21	Baicalin	C_21_H_18_O_11_	445.07751	11.446	87.1	33.50
22	Luteolin	C_15_H_10_O_6_	285.04062	11.773	87.2	23.75
23	Oroxylin A	C_16_H_12_O_5_	283.06125	11.788	90.4	24.75
24	Didymin	C_28_H_34_O_14_	593.18798	12.009	90.1	33.26
25	Wogonoside	C_22_H_20_O_11_	459.093	12.188	91.5	24.93
26	Hexahydrocurcumin	C_21_H_26_O_6_	373.1659	12.613	85.4	30.01
27	Hispidulin	C_16_H_12_O_6_	299.0562	12.707	89.2	36.79
28	Randaiol	C_15_H_14_O_3_	241.08724	13.291	80.8	152.77
29	Formononetin	C_16_H_12_O_4_	267.06628	13.61	88.7	49.00
30	Magnaldehyde D	C_16_H_14_O_3_	253.0871	14.129	84.2	81.10
31	Wogonin	C_16_H_12_O_5_	283.0611	14.291	89.9	23.53
32	Casticin	C_19_H_18_O_8_	373.0929	14.435	80.1	109.01

**Table 2:** Main components of QLXP in positive ion mode.

No.	Name	Formula	*m*/*z*	RT [min]	mzVault Best Match	Sample/ Control Ratio
1	Hordenine	C_10_H_15_NO	166.1224	2.271	86.3	31.83
2	Indoleacrylic acid	C_11_H_9_NO_2_	188.0704	5.299	84.4	35.78
3	Vicenin II	C_27_H_30_O_15_	595.16551	7.568	86	45.71
4	*N*-Methylcoclaurine	C_18_H_21_NO_3_	300.15912	7.754	89.6	21.01
5	Scutellarein	C_15_H_10_O_6_	287.05462	8.886	81.2	26.26
6	Calycosin-7-*O*-β-d-glucoside	C_22_H_22_O_10_	447.12807	8.968	82.9	25.81
7	Naringenin	C_15_H_12_O_5_	273.07539	9.718	89.1	38.50
8	Narirutin	C_27_H_32_O_14_	581.18599	9.718	89.8	35.54
9	Hesperetin	C_16_H_14_O_6_	303.08563	10.229	89.8	42.68
10	Hesperidin	C_28_H_34_O_15_	611.19639	10.231	89.4	34.81
11	Baicalein	C_15_H_10_O_5_	271.05936	10.828	83.4	23.90
12	Baicalin	C_21_H_18_O_11_	447.09099	10.829	80.5	24.28
13	Atractylenolide I	C_15_H_18_O_2_	231.1376	11.217	83.9	31.00
14	Formononetin	C_16_H_12_O_4_	269.08048	11.229	84.7	21.53
15	Jatrorrhizine	C_20_H_19_NO_4_	338.13782	11.237	90.1	22.20
16	Oroxylin A	C_16_H_12_O_5_	285.07512	11.791	83.1	27.36
17	Calycosin	C_16_H_12_O_5_	285.07516	11.931	92.5	22.71
18	Wogonin	C_16_H_12_O_5_	285.07495	12.195	82	24.61
19	Heterophyllin B	C_40_H_58_N_8_O_8_	779.44451	13.048	90.1	35.10
20	Isosinensetin	C_20_H_20_O_7_	373.12749	13.374	93.1	46.46
21	5-*O*-Demethylnobiletin	C_20_H_20_O_8_	389.12254	13.532	87.7	55.67
22	Sinensetin	C_20_H_20_O_7_	373.12687	13.853	90	48.41
23	6-Demethoxytangeretin	C_19_H_18_O_6_	343.11711	13.93	85.5	38.92
24	Nobiletin	C_21_H_22_O_8_	403.13754	14.363	92.8	30.35
25	Tangeretin	C_20_H_20_O_7_	373.1272	14.909	90.9	49.55
26	Curcumenol	C_15_H_22_O_2_	235.16861	14.924	91.1	31.01
27	Zederone	C_15_H_18_O_3_	247.13265	15.203	84.3	31.31
28	Dihydrotanshinone I	C_18_H_14_O_3_	279.10093	15.657	87	48.06
29	Cryptotanshinone	C_19_H_20_O_3_	297.1477	16.609	87.7	104.72
30	Tanshinone IIA	C_19_H_18_O_3_	295.13229	17.508	93	33.04

**Table 3:** Main components binding with ADAM17 in QLXP.

Name	Formula	*m*/*z*	RT [min]
Epiberberine	C_20_H_17_NO_4_	336.12263	12.735
Palmatine	C_21_H_21_NO_4_	352.15407	12.696
Jatrorrhizine	C_20_H_19_NO_4_	338.13866	11.872
(+)-Magnoflorine	C_20_H_23_NO_4_	342.16997	8.584
Berberrubine	C_19_H_15_NO_4_	322.10752	10.559
Demethyleneberberine	C_19_H_17_NO_4_	324.12316	10.732
Nobiletin	C_21_H_22_O_8_	403.13876	15.267
Sinensetin	C_20_H_20_O_7_	373.12831	14.714
Formononetin	C_16_H_12_O_4_	269.08093	14.401
Dehydroglaucine	C_21_H_23_NO_4_	354.16999	11.123
6-Demethoxytangeretin	C_19_H_18_O_6_	343.11792	15.312
Quinic acid	C_7_H_12_O_6_	191.05673	1.02
Oroxylin A-7-*O*-β-d-glucuronide	C_22_H_20_O_11_	459.09442	13.101
TF-Hesperidin	C_28_H_34_O_15_	609.184	11.383
Narirutin	C_27_H_32_O_14_	579.17336	10.778

**Table 4:** Molecular docking result of components binding ADAM17.

ChemSpider ID	Compound	XP GScore	MM-GBSA dG Bind (kcal/mol)
390871	Narirutin	−6.114	−34.20
65269	Jatrorrhizine	−2.447	−22.11
402990	Berberrubine	−2.200	−31.28
128491	Sinensetin	−1.373	−30.91

## Data Availability

The data presented in this study are available on request from the corresponding author. The data are not publicly available due to intellectual property protection.

## Abbreviations

GC	Gastric cancer
CAG	Chronic atrophic gastritis
IM	Intestinal metaplasia
TCM	Traditional Chinese medicine
RCT	Randomized controlled trial
QLXP	Qilianxiaopi formula
EMT	Epithelial–mesenchymal transition
TPP	Thermal proteome profiling
CETSA	Cellular thermal shift assay
BLI	Bio-layer interferometry
HPLC	High-performance liquid chromatography
LC-MS	Liquid chromatography-tandem mass spectrometry
NPARC	Nonparametric analysis of response curves
GO	Gene Ontology
PPI	Protein–protein interaction
HRP	Horseradish peroxidase
OD	Optical density
TMT	Tandem mass tag
Tm	Melting temperature
GSEA	Gene Set Enrichment Analysis
TACE	TNF-α-converting enzyme
ADAM	A Disintegrin And Metalloproteinase
